# Phylogeny more than plant height and leaf area explains variance in seed mass

**DOI:** 10.3389/fpls.2023.1266798

**Published:** 2023-11-16

**Authors:** Yingnan Wang, Yang Wang, Fei Yu, Xianfeng Yi

**Affiliations:** ^1^ School of Life Sciences, Qufu Normal University, Qufu, China; ^2^ College of Life Sciences, Henan Normal University, Xinxiang, China

**Keywords:** seed mass, plant height, leaf area, genome size, leaf N, growth form, phylogeny

## Abstract

Although variation in seed mass can be attributed to other plant functional traits such as plant height, leaf size, genome size, growth form, leaf N and phylogeny, until now, there has been little information on the relative contributions of these factors to variation in seed mass. We compiled data consisting of 1071 vascular plant species from the literature to quantify the relationships between seed mass, explanatory variables and phylogeny. Strong phylogenetic signals of these explanatory variables reflected inherited ancestral traits of the plant species. Without controlling phylogeny, growth form and leaf N are associated with seed mass. However, this association disappeared when accounting for phylogeny. Plant height, leaf area, and genome size showed consistent positive relationship with seed mass irrespective of phylogeny. Using phylogenetic partial R^2^s model, phylogeny explained 50.89% of the variance in seed mass, much more than plant height, leaf area, genome size, leaf N, and growth form explaining only 7.39%, 0.58%, 1.85%, 0.06% and 0.09%, respectively. Therefore, future ecological work investigating the evolution of seed size should be cautious given that phylogeny is the best overall predictor for seed mass. Our study provides a novel avenue for clarifying variation in functional traits across plant species, improving our better understanding of global patterns in plant traits.

## Introduction

Seed mass, a key ecological trait that affects many aspects of plant ecology (Moles et al., 2005a; Moles et al., 2005b; Mason et al., 2008), has great influences on the regeneration strategies of plants, including seed output for a given amount of energy, seed dispersal and seedling survival (Leishman et al., 2000; Chen et al., 2022; Cui et al., 2023). Variation in seed mass reflects the fundamental trade-off between seed number and seed mass (Henery and Westoby, 2001) and between seed mass and persistence in the seed bank (Thompson et al., 1993). An increasing body of evidence has shown that large-seeded species produce fewer seeds than those bearing small seeds (Henery and Westoby, 2001; Moles et al., 2004). Compared to small-seeded species, large-seeded species are more likely to produce large seedlings that are supposed to survive better than small seedlings under a variety of hazardous environments (Armstrong and Westoby, 1993; Leishman and Westoby, 1994a; Leishman and Westoby, 1994b; Burke and Grime, 1996; Westoby et al., 1996; Harms and Dalling, 1997; Leishman et al., 2000; Dalling and Hubbell, 2002; Westoby et al., 2002; Moles and Westoby, 2004; Dainese and Sitzia, 2013). Seed masses of present-day species have been observed to range over 11.5 orders of magnitude, from the 0.0001-mg dust-like seeds of orchids to the 20-kg seeds of the double coconut (Leishman et al., 1995). It has been recognized, therefore, that understanding the influencing factors that drive changes in seed mass can help elucidate plant ecological history (e.g., Moles et al., 2005b).

To date, there have been many potential explanations for the variation in seed mass. The leaf-height-seed (LHS) scheme proposed by Westoby (1998), which encompasses variation in a number of correlated plant characteristics (leaf area, plant height, and seed size), has been used to quantify the strategy to explain the variation in seed mass in response to the other two functional traits. At the same time, seed mass could be correlated with other basic life-history traits, such as growth form, genome size, leaf N and other potential explanatory variables. Moreover, seed mass might be best predicted by phylogeny, showing phylogenetic conservatism in evolution of seed size. Although knowledge is available for the effect of single trait on seed size variation, incorporating multiple ones is expected to illustrate to which degree seed size will be influenced by the potential explanatory variables.

As a crucial component of a plant species’ ecological strategy (Westoby, 1998), plant height not only determines a plant’s ability to compete for light but also a species’ carbon gain strategy, which is supposed to play an important role in another life-history trait, seed mass. A pioneering study by Levin (1974) found that the mean seed mass of 832 plant species increase along the growth form height gradient of herbs, shrubs, vines, shrubby trees, and trees. Leishman et al. (1995) showed that seed masses are consistently correlated with plant height across 1659 species, representing a worldwide flora. Similar pattern of the correlation between seed mass and plant height was observed by Moles et al. (2004) and Carly et al. (2009). However, Grime et al. (1997) found no significant correlation between plant height and seed mass across 43 common British species. Thompson and Rabinowitz (1989) analyzed 816 plant species around Sheffield and found significant relationships between seed mass and plant height within some families, but not in other taxa. In a southeastern Sweden flora, seed mass was only marginally correlated with plant height of 126 species (Bolmgren and Cowan, 2007). Rees (1996) analyzed 382 species of Sheffield flora and found that the relationship between seed mass and plant height is inconsistent and dependent on dispersal modes. Although plant height has been considered one of the strongest correlates of seed mass (Leishman et al., 1995; Moles et al., 2004), much uncertainty still remains to be tackled, possibly because of sampling and taxonomic breadth in earlier literature.

As the main organ of plants that contributes to photosynthesis, leaves act as a key determinant of the amount of energy available for reproduction (Wright et al., 2004). Although leaves may vary in their traits (e.g., area and N nutrition) in response to growing conditions (Givnish, 1987; Witkowski and Lamont, 1991; Ackerly and Reich, 1999; Cornelissen et al., 2003; McDonald et al., 2003; Xu et al., 2009; Milla and Reich, 2011), a strong connection between total leaf mass and net annual reproductive biomass has been observed (Niklas and Enquist, 2002). Therefore, the ecological significance of leaf traits may relate to resource capture in productive organs, implying that leaf area and seed mass should be positively correlated (Westoby and Wright, 2003). Leaf area was found to be positively correlated to seed mass across plant species in South Africa, England, Spain and northern Arizona, USA (Midgley and Bond, 1989; Laughlin et al., 2010; Hodgson et al., 2017). In contrast, Cornelissen (1999) showed a non-linear relationship between leaf area and seed mass of 58 woody species from Europe. Recently, Santini et al. (2017) showed that the triangular relationship also holds for 401 annual plants belonging to 37 families from the United Kingdom. However, Westoby and Wright (2003) failed to find the triangular relationship between leaf area and seed mass as reported by Cornelissen (1999), indicating that the pattern seems not universal between seed mass and leaf area.

In addition, seed mass is not independent of growth form, which is often a predictor of other plant traits (Moles et al., 2005a, b). Plant growth form, like seed mass, may also be phylogenetically constrained (Li et al., 2017). Evidence has shown that woody plants are more likely to have larger seeds, while non-woody species are more likely to produce small seeds (Jurado et al., 1991). Therefore, the phylogenetic constrains of plant growth form might have an indirect impact on the variations in seed mass. Furthermore, genome size appears to be one of the most studied factors that are related to variations in seed mass. The relationship between genome size and seed mass has been shown to vary among life forms in flowering seed plants (Beaulieu et al., 2007). Carta et al. (2022) found that species with very large genome sizes never had small seeds. Therefore, apart from the influence of plant height and leaf area, phylogeny, growth form, and genome size may also contribute to seed mass variations.

Phylogenetic conservatism in plant traits has been well studied (Wiens et al., 2010; Cornwell et al., 2014; Tozer et al., 2015) and such studies are helping to illuminate the role of the evolutionary past in determining the characteristics of species. Seed mass has been accepted as an ecologically important trait phylogenetically constrained within local floras. This may also be true for plant height and leaf area. Therefore, it would provide deeper insight into the variations in plant traits associated with phylogeny, before analyzing relationships between seed mass and other plant ecological attributes, e.g., growth form, plant height, and leaf area. However, the potential influence of phylogeny on the leaf-height-seed (LHS) plant ecology strategy scheme has not previously been well evaluated (Cornelissen, 1999; Laughlin et al., 2010; Hodgson et al., 2017).

Previous data on the relationship between plant traits has been published across the world (Lord et al., 1995; Kang and Primack, 1999; Zhang et al., 2004; Vandelook et al., 2018). The rapid accumulation of databases on plant traits provides us an ideal opportunity to illustrate a general pattern of the relationship between plant traits (Salguero-Gómez et al., 2015; Kattge et al., 2020; Carta et al., 2022), which helps us to have a better understanding of the leaf-height-seed (LHS) plant ecology strategy scheme. In the present study, we first used phylogenetic partial R^2^s (Ives, 2019) to tease apart the effects of multiple plant traits (plant height, leaf area, genome size, growth form and leaf N) and phylogeny, to quantify extent to which they contribute to variations in seed mass of plant species when each predictor variable and the phylogeny is removed one-by-one.

## Materials and methods

### Data collection

Plant traits were derived from the large currently available databases TRY plant trait database (Kattge et al., 2020) and Plant DNA C-values Database (Royal Botanic Gardens Kew, 2022) as well as published literature (Westoby and Wright, 2003; Díaz et al., 2016; Santini et al., 2017). Raw data collected from various sources was cleaned and curated. For example, mean value was calculated if a single species has multiple trait values. Plant traits included: 1) seed mass (mg seed^-1^), maximum plant height (m), genome size (1C, pg), leaf area (mm^2^), leaf N (mg/g), and growth form. The growth form was split into two functional groups: “woody” and “non-woody” because sample size was not sufficient for extracting more detailed growth form classes. We took advantage of big data and compiled a globally distributed dataset containing 1071 vascular plant species, covering 553 genera, 136 families, 52 orders with information of seed mass, maximum plant height, genome size, leaf area, and leaf N. Our final data base for the main analysis contained 404 woody and 667 non-woody species with known trait values, representing 0.3% vascular plants in the world. In total, 1002 angiosperms were analyzed together with 69 gymnosperms in which different LHS strategy has been observed. Following Westoby (1998), all variables were log10-transformed prior to analysis to correct for skewness in trait distributions because trait values can vary by several orders of magnitude, and are often log normally distributed between species.

### Phylogenetical signal

Phylogenetic signal in seed mass, plant height, leaf area, growth form and leaf N was calculated using a phylogenetic tree (GBOTB.extended.tre) obtained by pruning the largest phylogeny for vascular plants so far, containing 10587 genera and 74533 vascular plant species (Zanne et al., 2014; Smith and Brown, 2018). The R package ‘V. PhyloMaker’ was used because it can generate very large phylogenies for vascular plants at a relatively fast speed (Jin and Qian, 2019). Species names in this study were checked and standardized according to the Plant List v.1.1 (http://www.theplantlist.org/).

Pagel’s lambda (λ) estimates the strength of phylogenetic signal in a continuous trait, therefore, we calculated Pagel’s λ to quantitatively estimate if the similarity of seed mass, plant height, genome size, leaf area, and leaf N among species is correlated with the phylogenetic similarity of plant species. We utilized the canned randomizations by running the package ‘phytools’ (Revell, 2012) in R to test for the significance of λ. In our study, Pagel’s λ can range from 0 to 1, with a λ of 0 indicating no phylogenetic signal and whereas a λ of 1 indicating the strongest phylogenetic signal (Pagel, 1999).

We tested the strength of the phylogenetic signal in growth form using the D statistic that is for binary traits (Fritz and Purvis, 2010), using the package ‘caper’ in R. Growth form of the 1071 species is supposed to come from the time of their independent evolution if the D is not significantly different from 0 (P_Brownian_ > 0.05). Whereas, if D value is equal to or not significantly different from 1 (P_random_ > 0.05), which indicates that the interspecific differences in growth form are distributed randomly across a phylogenetic tree.

### Statistical analysis

All analyses were conducted in R (R Development Core Team, 2021). As plant traits vary with growth form, we analyzed for differences in plant traits between woody and non-woody species. We employed the general linear model to detect the differences in seed mass, plant height, genome size, leaf area, leaf N between plant species with different growth forms (woody vs non-woody). We also constructed generalized linear model (GLM) to see the association between seed mass, plant height, growth form, genome size, leaf area and leaf N across all plant species and groups, with the seed mass as dependent variable and other plant traits as independent variables. To investigate which plant traits were more important to variations in seed mass across plant species, we applied a multi-variable phylogenetic generalized linear mixed model (PGLMM) to incorporate phylogenetic information and then correct for phylogenetic effects among species, as closely related organisms are more likely to share similar biological traits. We used a Gaussian distribution with phylogenetic trees, implemented in the R packages ‘phyr’ and ‘ape’ (Paradis and Schliep, 2019; Li et al., 2020). We considered plant height, leaf area, genome size, growth form and leaf N as predictor variables, seed mass as the response variable and phylogeny as a random intercept.

To tease apart the relative contributions of plant traits and phylogeny to the variation in seed mass of the plant species, we used partial R^2^s for the logistic regression model (Ives, 2019) implemented by the R package “rr2” (Ives and Li, 2018). The partial R^2^_lik_ for each factor was calculated by comparing the full model with reduced models in which a given factor was removed, and measuring the consequent reduction in the likelihood (Wang et al., 2022).

## Results

**Figure 1:**
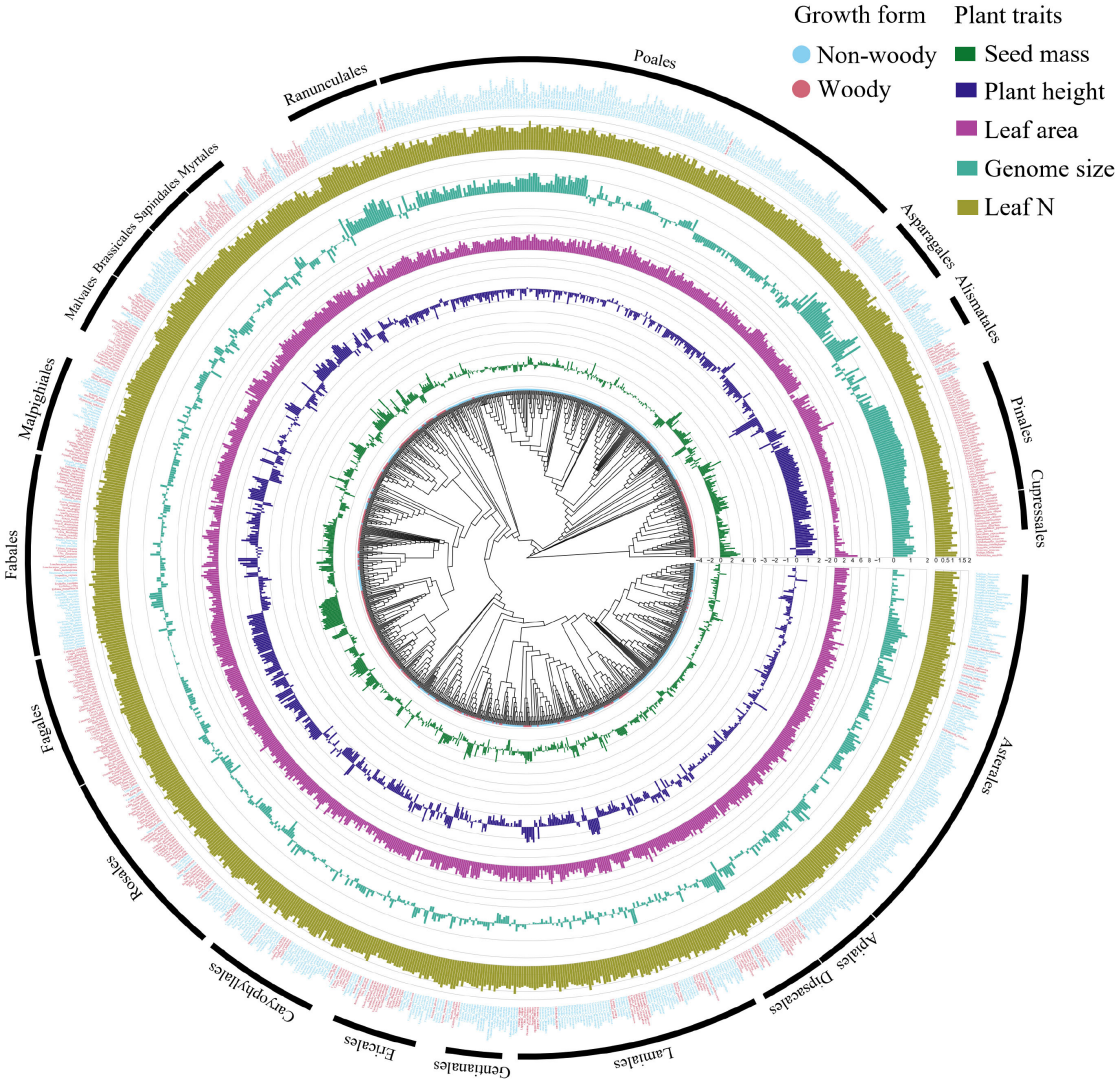
Plant traits (seed mass, plant height, leaf area, genome size, leaf N, and growth form) mapped onto a plant phylogeny. Note that data were log 10-transformed prior to mapping. Note: orders covering > 10 species are shown outside the phylogeny tree.

**Figure 2:**
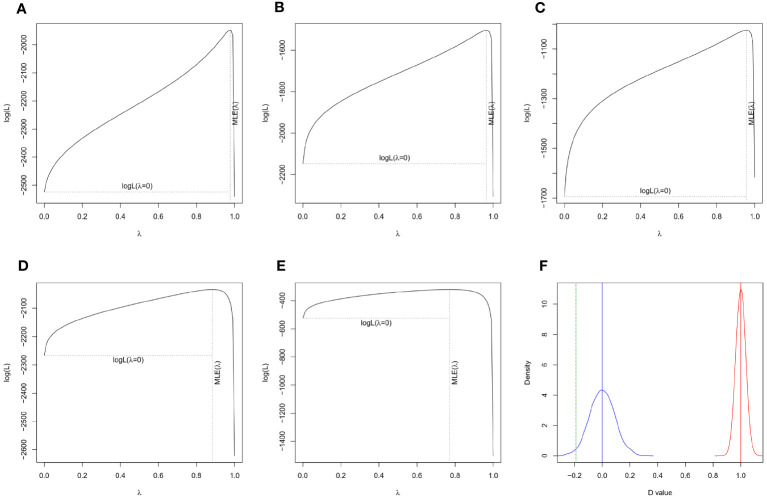
Tests of phylogenetic signal in plant traits. A statistically significant phylogenetic signal was detected in seed mass (**A**, λ = 0.976, P < 0.001), plant height (**B**, λ = 0.964, P < 0.001), genome size (**D**, λ = 0.956, P < 0.001), leaf area (**C**, λ = 0.883, P < 0.001), leaf N (**E**, λ = 0.771, P < 0.001), and growth form (**F**, D = -0.148, P_random_ = 0, P_Brownian_ = 0.982) of 1071 plant species.

By analyzing worldwide variation in several plant traits, we found strong and statistically significant phylogenetic signal of seed mass (λ = 0.976, P < 0.001), plant height (λ = 0.964, P < 0.001), genome size (λ = 0.956, P < 0.001), leaf area (λ = 0.883, P < 0.001), leaf N (λ = 0.771, P < 0.001) and growth form (D = -0.190, P_random_ = 0, P_Brownian_ = 0.982) across the plant species (**Figures 1**, **2**), showing that plant traits covary in direct proportion to their shared evolutionary history.

**Figure 3:**
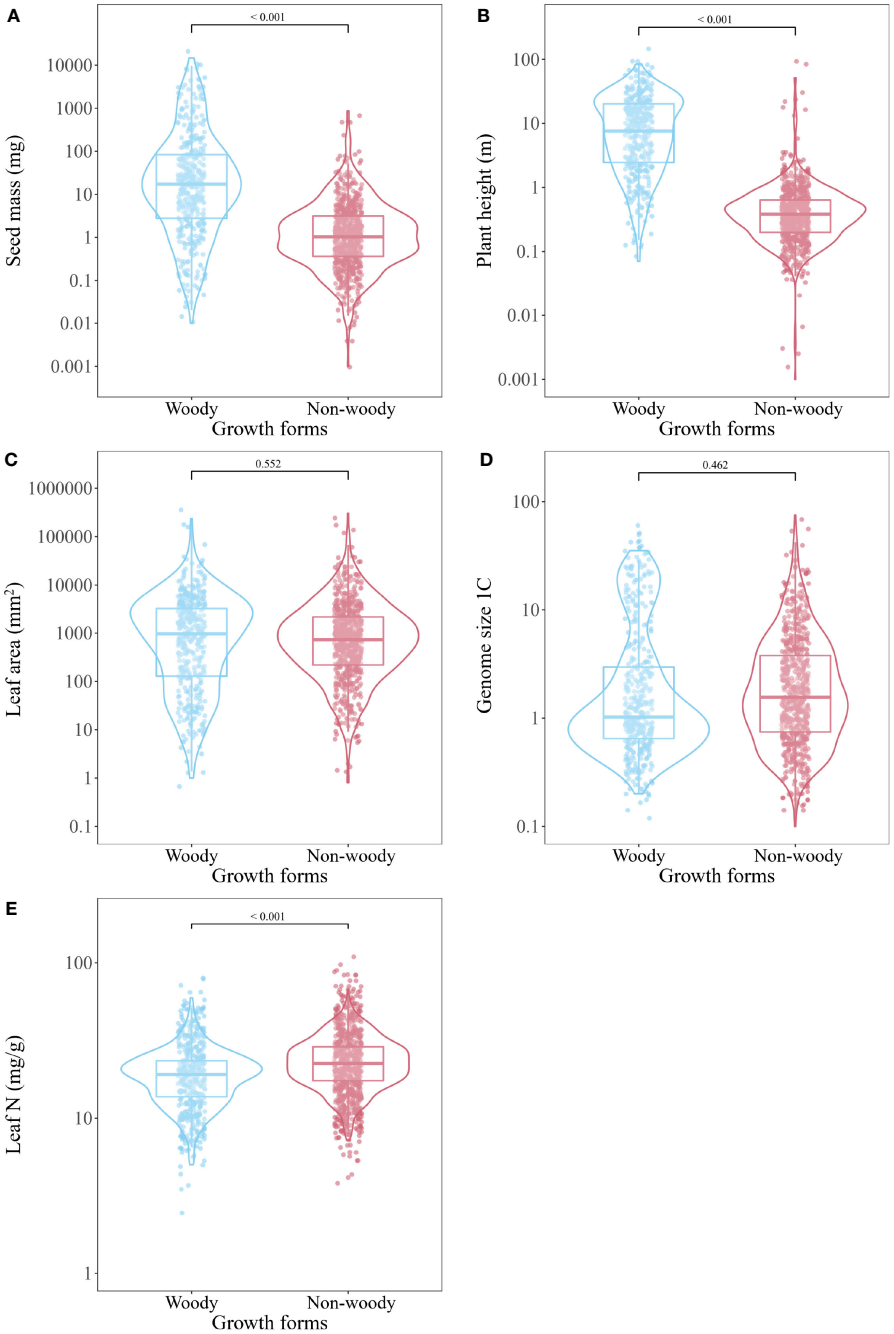
Comparison of seed mass **(A)**, plant height **(B)**, leaf area **(C)**, genome size **(D)** and leaf N **(E)** between plant species with different growth forms (woody vs non-woody) derived from general linear model (GLM).

General linear model showed that seed mass and plant height were higher in the woody plants than in the non-woody species (t = -6.676, P < 0.001; t = -38.42, P < 0.001; **Figures 3A, B**). Although there was a trend for woody plants to have larger genome size and leaf area than non-woody species, this was not significant (t = 0.735, P = 0.462; t = 0.595, P = 0.552; **Figures 3C, D**). However, leaf N was lower in the woody plants than in the non-woody species (t = 8.782, P < 0.001; **Figure 3E**). We found no difference in seed mass between angiosperms and gymnosperms, but a significant difference in leaf N between Leguminosae and other families (P < 0.05).

Without controlling phylogeny, we identified statistically significant relationships between seed mass and plant height across 1071 species based on multi-variable generalized linear model (t = 3.299, P = 0.001; **Table 1**). Although seed mass and leaf area were positively correlated (t = 6.961, P < 0.001; **Table 1**), we detected significant interactive effect of plant height and leaf area on variations in seed mass (t = 2.204, P = 0.028; **Table 1**). Significant relationship was found between seed mass and genome size (t = 5.034, P < 0.001; **Table 1**) but not between seed mass and leaf N in the absence of phylogeny (t = 1.646, P = 0.099; **Table 1**). Growth form, however, well predicted variations in seed mass across the plant species (t = 5.784, P < 0.001; **Table 1**).

**Table 1:** Multivariate phylogenetic generalized linear mixed model (PGLMM) and generalized linear model (GLM) constructed with seed mass of the 1071 species as response variable.

Model	AIC	Predictor variable	Estimate (SE)	t	*P*
GLM	2731.5	Intercept	-0.822 (0.233)	-3.522	< 0.001
		Plant height	0.330 (0.100)	3.299	0.001
		Leaf area	0.231 (0.033)	6.961	< 0.001
		Genome size	0.281 (0.056)	5.034	< 0.001
		Leaf N	0.271 (0.165)	1.646	0.099
		Growth form	0.527 (0.091)	5.784	< 0.001
		Plant height*Leaf area	0.081 (0.037)	2.204	0.028

Model	AIC	Predictor variable	Estimate (SE)	z	*P*
PGLMM	1976.6	Intercept	0.614 (0.893)	0.688	0.492
		Plant height	0.409 (0.096)	4.268	< 0.001
		Leaf area	0.071 (0.028)	2.549	0.011
		Genome size	0.304 (0.067)	4.514	< 0.001
		Leaf N	0.103 (0.121)	0.850	0.395
		Growth form	0.075 (0.089)	0.838	0.402
		Plant height*Leaf area	0.015 (0.035)	0.437	0.662

In analysis that controlled for the phylogeny, plant height, leaf area, and genome size alone appeared to be a reliable predictor of variations in seed mass (z = 4.268, P < 0.001; z = 2.549, P = 0.011; z = 4.514, P < 0.001; **Table 1**). After correcting for phylogenetic effects, growth form and leaf N failed to predict variations in seed mass (z = 0.838, P = 0.402; z = 0.850, P = 0.395; **Table 1**). There was no significant interactive effect of plant height and leaf area on seed mass in the presence of phylogenetic considerations (z = 0.437, P = 0.662; **Table 1**).

**Figure 4:**
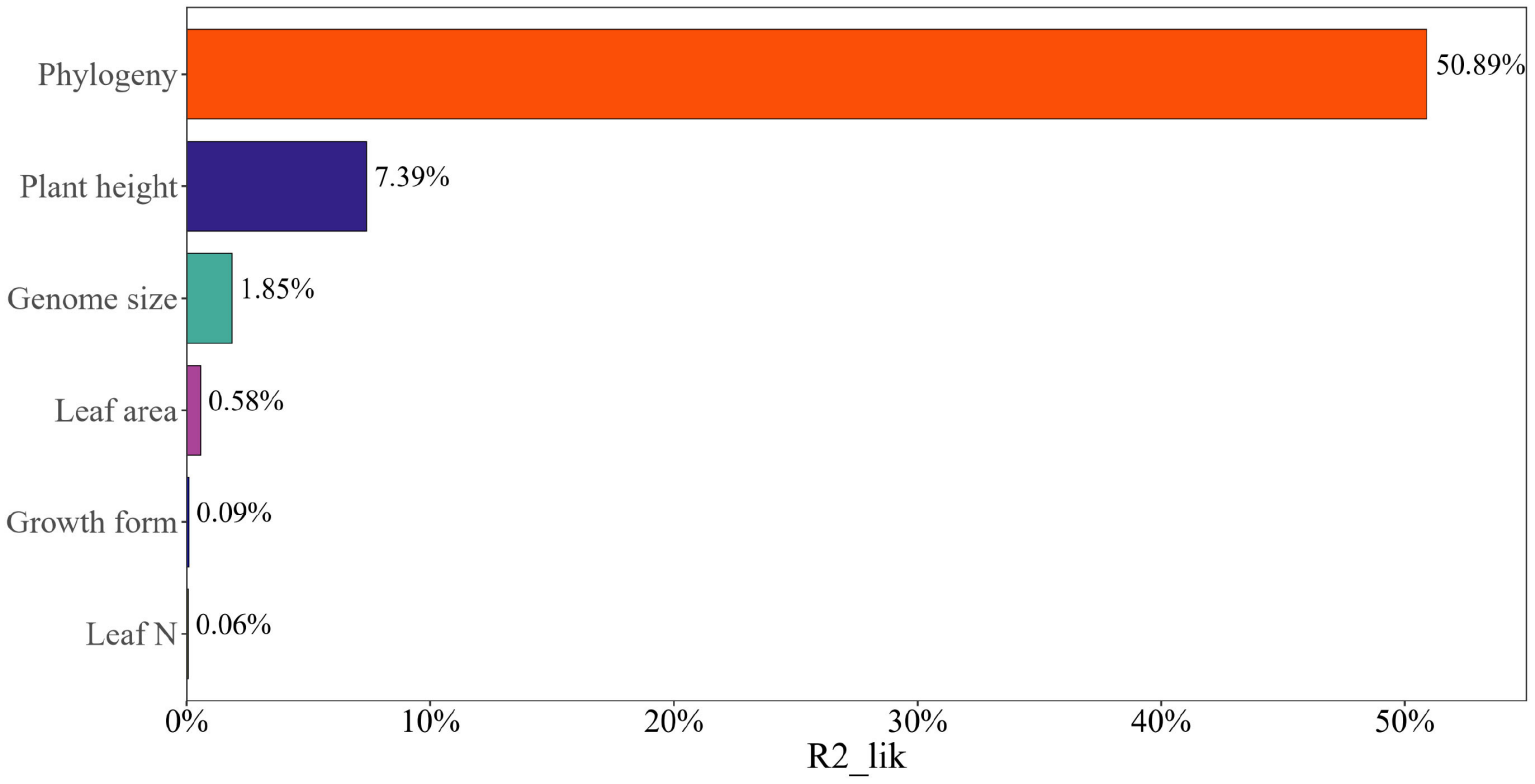
The relative contribution of different factors to the variation in seed mass using partial R^2^s for the logistic regression model.

Phylogeny explained the vast majority of seed mass variation across the plant species (partial R^2^_lik_ = 50.89%, ΔlogLik = 380.8, P < 0.001), while plant height, leaf area, genome size explained a minority of variation (R^2^_lik_ = 7.39%, ΔlogLik = 41.1, P < 0.001; R^2^_lik_ = 0.58%, ΔlogLik = 3.1, P = 0.01; R^2^_lik_ = 1.85%, ΔlogLik = 10.0, P < 0.001; **Figure 4**). Leaf N and growth form failed to explain the variance in seed mass (R^2^_lik_ = 0.06%, ΔlogLik = 0.3, P = 0.41; R^2^_lik_ = 0.09%, ΔlogLik = 0.5, P = 0.33; **Figure 4**).

## Discussion

By conducting an analysis of a collated dataset of 1071 plant species, our results suggest phylogenetic clustering for the majority of plant traits tested in this study, i.e., seed mass, plant height, leaf area, genome size, and leaf N, as observed in previous studies (Moles et al., 2005b; Swenson and Enquist, 2009; Davies et al., 2013; Wang et al., 2022). This is most likely due to the similar traits of phylogenetically closely related species rather than the similarity of traits at higher taxonomic levels, e.g., genus and family. Apart from phylogenetic signal of these traits, we showed that growth form of the 1071 species was not randomly distributed but followed a Brownian model of trait evolution, indicating that the closely related species are more likely to have similar growth form than might be expected by chance (Kerkhoff et al., 2006). As geographic distribution of plant species is greatly shaped by growth form (Xu et al., 2018; Zhao et al., 2018), identifying the phylogenetic distribution of woody versus non-woody species will advance our knowledge of terrestrial plant distribution in various ecosystems.

Previous studies have looked at the patterns of associations of seed mass with other plant traits (Westoby, 1998; Guo et al., 2010; Santini et al., 2017). By controlling phylogeny, plant height appears to be a reliable predictor of seed mass across species based on the results of PGLMM. Although dispersal mode and growth form may modify the pattern of association of plant height with seed mass (Thompson and Rabinowitz, 1989; Leishman and Westoby, 1994c; Leishman et al., 1995), we found consistent positive correlation between seed mass and plant height. The positive relationship between seed mass and plant height may facilitate long-distance seed dispersal because seed dispersal distance is more strongly correlated with plant height than with seed mass (Thomson et al., 2011).

Plant height is also closely related to leaf area (Falster and Westoby, 2003), therefore positive relationships between leaf area and plant height are likely to lead to a positive correlation between seed mass and leaf area. In our study, there were also consistent and positive correlations between seed mass and leaf area, suggesting that the correlations between leaf area and seed mass are conserved across life-forms. These patterns accord well with independently gathered data on the relationship between seed mass and leaf area both in the woody and annual species (Senn et al., 1992; Niinemets and Kull, 1994; Cornelissen, 1999; Santini et al., 2017).

The role of the relationship between genome size and seed mass has gained much less attention over the two decades (Moles et al., 2005a, b; Beaulieu et al., 2007). Despite several studies that found a quadratic relationship between genome size and seed mass (Beaulieu et al., 2007; Knight and Beaulieu, 2008; Krahulcová et al., 2017), our GLM and PGLMM models showed positive association between genome size and seed mass across 1017 species. To understand the forces shaping the evolution of seed mass, we will also need to consider other plant traits, such as leaf N and growth form. Without controlling phylogeny, seed size was associated with growth form and woody plants tended to have larger seeds than smaller herbaceous plants, possibly due to the larger height of woody plants than of herbaceous species (Jurado et al., 1991). However, incorporating phylogenetic affiliations into the model failed to detect the clear association between seed mass and growth form across the plant species, indicating that growth form is phylogenetically conserved. This finding may not be in agreement with the observation that variations in seed mass are consistently associated with those in growth form (Moles et al., 2005a).

Despite the strong phylogenetic signal in several plant traits, our study successfully teased apart the relative contributions of phylogeny, plant height, leaf area, genome size, leaf N and growth form on explaining variations in seed mass across the plant species. We first showed that phylogeny had much more power to explain variations in seed mass than did other plant traits, whereas plant height, leaf area and genome size only explained the minority of these variations although the leaf-height-seed (LHS) scheme states that plant height and leaf area are closely correlated with seed mass (Westoby, 1998). Growth form and leaf N explained little variation in seed mass, reflecting the main effect of phylogeny on affecting seed mass variation. Therefore, our study suggests that divergences in seed mass have been more closely correlated with phylogeny than with divergences in other plant traits. If this pattern holds equally for plants of different taxa, investigation on the correlations between plant traits should not ignore the contribution of phylogeny.

We admit that there are some limitations to our study. Although we acknowledge that the trait data is inherently limited when multiple functional traits of plants are included, 1071 species investigated in our study account for a very small minority of total global vascular plant species, which is unable to completely represent the full diversity of seed plants. In addition, species analyzed in our study are mainly included in families such as Compositae, Lamiaceae, Plantaginaceae, Leguminosae, Rosaceae, Fagaceae, Ranunculaceae, Poaceae, Cyperaceae, and Pinaceae, whereas rarely found in Aristolochiaceae, Chloranthaceae, Schisandraceae, Nymphaeaceae, Liliaceae, Pontederiaceae, and Flagellariaceae, which in turn results in many taxa lacking in biodiversity-rich areas such as Africa. Therefore, some potential bias will be present due to plant species over-sampled or under-sampled in our study. Failure to include masses of plant taxa in the model will not provide an unbiased pattern of seed mass variation. Moreover, a global dataset without considering the geographic heterogeneity of the 1071 vascular species were analyzed using the partial R^2^s for the logistic regression model, which may overestimate the contributions of phylogeny and other plant traits to variations in seed mass across plant species.

Taken together, our results indicate that although various plant traits (seed mass, plant height, leaf area, genome size, leaf N and growth form) are phylogenetically conserved and closely correlated, phylogeny appears to explain variations in seed mass better than other explanatory variables. Based on the partial R^2^s for the logistic regression model, our results provide solid evidence that phylogeny is the best overall predictor for seed mass, warning that future ecological work on the correlations of seed size with other plant traits and external variables should be cautious. The strong phylogenetic signals of plant traits in this study provide an implication that the external, abiotic, climatological factors are potentially less important for determining variations in plant traits, though variation in plant traits can be partially explained by the habitat characteristics. It can be expected that the observed patterns in our study will be true for a majority of vascular plants within certain ecosystems because 533 genera belonging to 136 families were included in our analyses, representing a worldwide flora. Our results may also suggest a tight relationship between plant phylogeny and the geographic distributions because of similar selection pressures experienced by species from a common ancestor in similar habitats (Chen et al., 2022). Given that seed mass and plant height are so tightly linked with reproductive potential and dispersal (Leishman et al., 2000; Thomson et al., 2011; Hou, et al., 2021; Wang and Yi, 2022), the phylogenetic relatedness of plant species may influence their distribution range size (Moles et al., 2005b). Therefore, our work is expected to open the door to further investigate the contributions of phylogeny and explanatory attributes to the variation in given plant traits.

## Data availability statement

The datasets presented in this study can be found in online repositories. The names of the repository/repositories and accession number(s) can be found in the article/**Supplementary Material**.

## Author contributions

YNW: Data curation, Formal Analysis, Writing – original draft. YW: Data curation, Formal Analysis, Methodology, Writing – original draft. FY: Conceptualization, Data curation, Supervision, Writing – original draft. XY: Conceptualization, Funding acquisition, Supervision, Writing – original draft, Writing – review and editing.
